# The Olfactory Receptor Gene Product, OR5H2, Modulates Endometrial Cancer Cells Proliferation via Interaction with the IGF1 Signaling Pathway

**DOI:** 10.3390/cells10061483

**Published:** 2021-06-12

**Authors:** Rand Shibel, Rive Sarfstein, Karthik Nagaraj, Lena Lapkina-Gendler, Zvi Laron, Manisha Dixit, Shoshana Yakar, Haim Werner

**Affiliations:** 1 Department of Human Molecular Genetics and Biochemistry, Sackler School of Medicine, Tel Aviv University, Tel Aviv 69978, Israel; randshibel@mail.tau.ac.il (R.S.); rives@tauex.tau.ac.il (R.S.); mailkartz@gmail.com (K.N.); lenalapkina@gmail.com (L.L.-G.); 2 Endocrinology and Diabetes Research Unit, Schneider Children’s Medical Center, Petah Tikva 49292, Israel; laronz@clalit.org.il; 3 David B. Kriser Dental Center, Department of Basic Science and Craniofacial Biology, New York University College of Dentistry, New York, NY 10010-4086, USA; dixitm01@nyu.edu (M.D.); sy1007@nyu.edu (S.Y.)

**Keywords:** insulin-like growth factor-1 (IGF1), IGF1 receptor, olfactory receptors, OR5H2, Laron syndrome, endometrial cancer

## Abstract

Endometrial cancer is the most common gynecologic malignancy in Western countries. The insulin-like growth factor-1 (IGF1) axis has an important role in endometrial cancer biology and emerged as a promising therapeutic target in oncology. However, there is an urgent need to identify biomarkers that may help in patient stratification and prognosis. Laron syndrome (LS) is a type of dwarfism that results from the mutation of the growth hormone receptor (GHR) gene, leading to congenital IGF1 deficiency. While high circulating IGF1 is regarded as a risk factor in cancer, epidemiological studies have shown that LS patients are protected from cancer development. Recent genome-wide profilings conducted on LS-derived lymphoblastoid cells led to the identification of a series of genes whose over- or under-representation in this condition might be mechanistically linked to cancer protection. The olfactory receptor 5 subfamily H member 2 (*OR5H2*) was the top downregulated gene in LS, its expression level being 5.8-fold lower than in the control cells. In addition to their typical role in the olfactory epithelium, olfactory receptors (ORs) are expressed in multiple tissues and play non-classical roles in various pathologies, including cancer. The aim of our study was to investigate the regulation of *OR5H2* gene expression by IGF1 in endometrial cancer. Data showed that IGF1 and insulin stimulate OR5H2 mRNA and the protein levels in uterine cancer cell lines expressing either a wild-type or a mutant p53. OR5H2 silencing led to IGF1R downregulation, with ensuing reductions in the downstream cytoplasmic mediators. In addition, OR5H2 knockdown reduced the proliferation rate and cell cycle progression. Analyses of olfr196 (the mouse orthologue of OR5H2) mRNA expression in animal models of GHR deficiency or GH overexpression corroborated the human data. In summary, OR5H2 emerged as a novel target for positive regulation by IGF1, with potential relevance in endometrial cancer.

## 1. Introduction

The growth hormone (GH)-insulin-like growth factor-1 (IGF1) endocrine axis is the main regulator of growth and development throughout life [1,2,3]. GH binding to the GH receptor (GHR) leads to receptor dimerization and the phosphorylation of JAK2, with the ensuing stimulation of IGF1 biosynthesis [4,5]. Circulating IGF1 is predominantly produced by the liver, although additional organs (e.g., brain, kidney, stomach, etc.) display the enzymatic machinery required to produce IGF1. The biological actions of IGF1 are mediated via the activation of the IGF1 receptor (IGF1R), a ubiquitously expressed tyrosine kinase-containing cell-surface receptor [6,7,8]. The IGF1R signals mitogenic, prosurvival and antiapoptotic actions and is regarded as a key player in a number of pathological conditions, including cancer.

Growth retardation in children is considered a multifactorial pathology [9,10]. Prenatal IGF1 expression is GH-independent, though it becomes dependent on GH secretion shortly before birth and remains GH-dependent during postnatal life. Laron syndrome (LS) is the best characterized entity under the spectrum of the congenital IGF1 deficiencies [11,12]. LS is caused by the mutation or deletion of the *GHR* gene, or post-receptor pathways, leading to congenital IGF1 deficiency and dwarfism [13]. The typical features of LS are obesity, typical face, high basal serum GH and low to undetectable IGF1, unresponsive to administration of GH [14]. While high endocrine IGF1 is linked to increased cancer risk, epidemiological studies have shown that LS patients are protected from cancer development [15,16,17,18].

Genome-wide analyses aimed at identifying genes and signaling pathways that are differentially represented in LS and that might account for cancer protection were recently performed. Profilings were conducted on immortalized lymphoblastoids from patients and healthy controls of the same ethnic groups [19]. Major differences between LS and the controls were noticed in pathways associated with cell cycle dynamics, autophagy and apoptosis [20]. Furthermore, ~17% of the genes shown to be differentially expressed in LS cells were members of the G protein-coupled receptor (GPCR) superfamily. A particular member of this family, the olfactory receptor 5 subfamily H member 2 (*OR5H2*), was the top downregulated gene in LS, its expression level being 5.8-fold lower than in the controls (*p* = 0.0018). No previous studies have identified a functional linkage between OR5H2 and the IGF1 pathway.

Olfactory receptors (ORs) are a large group of GPCRs, including approximately 380 functional genes [21]. ORs are predominantly found in the olfactory epithelium; however, many ORs are ectopically expressed in multiple tissues and organs, including the testes, lung, intestine, skin, heart and blood [22]. Ectopic ORs are involved in the regulation of a number of physiological processes such as glucose homeostasis, systemic blood pressure, etc. [23]. In addition, ORs play key roles in certain pathologies, including cancer, and have been validated as biomarkers in prostate tumors [24,25,26,27,28]. Of relevance, specific ORs were correlated with a series of breast cancer features [29].

Endometrial cancer is the most common gynecologic malignancy in Western countries. Cases are classified into two main categories according to their clinical, morphological and molecular features [30,31]. Type I tumors are usually estrogen-dependent, low-grade neoplasms, with an endometrioid and a well-differentiated morphology. Type II tumors are generally associated with p53 mutations, are not associated with exposure to estrogens and display a less differentiated phenotype [32]. While Type I tumors typically correlate with a relatively good prognosis, Type II tumors entail, in most cases, a poor prognosis. Uterine serous endometrial carcinoma (USC) constitutes the predominant histological category among Type II tumors [33]. IGF1 has a major role in uterine development and function. Cyclic changes in IGF1 expression and action play an important role in regulating the transition of the premenopausal endometrium through proliferative, secretory and menstrual cycles [34,35].

In view of the important roles of the IGF1 signaling axis in endometrial cancer biology, and given our recent identification of the *OR5H2* gene as a candidate downstream target for IGF1 action, we investigated in the present paper the regulation of *OR5H2* gene expression by IGF1 in endometrial cancer cells. The rationale for this research stems from the fact that the IGF1R emerged as a promising therapeutic target in oncology and there is an urgent need to identify tumor biomarkers that can predict therapy success. In addition, given the important role of tumor suppressor p53 in endometrial cancer etiology, we assessed the impact of the p53 mutational status on *OR5H2* regulation by using cell lines expressing a wild-type or a mutant p53. Finally, *olfr196* (the mouse orthologue of *OR5H2)* gene expression was investigated in GHR knock-out mice, an animal model of IGF1 deficiency, and in GH transgenic mice. Our data demonstrate that the *OR5H2* gene constitutes a novel target for IGF1 action in endometrial cancer. The potential clinical relevance of the present findings merits further investigation.

## 2. Materials and Methods

### 2.1. Cell Cultures and Treatments

The human uterine serous carcinoma (USC) cell lines USPC-1 and USPC-2 were employed in this study [36]. Cell lines were derived from USC patients who experienced rapid tumor progression during adjuvant chemotherapy after primary surgical debulking [37]. Cells were kindly provided by Dr. A. D. Santin, Yale University, New Haven, CT, USA. Cells were maintained in an RPMI-1640 medium supplemented with 10% fetal bovine serum (FBS), 2 mM glutamine, 100 units/mL penicillin, 100 mg/mL streptomycin and 5.6 mg/mL amphotericin B. Epstein–Barr virus-immortalized human lymphoblastoid cell lines from LS patients and healthy controls were obtained from the National Laboratory for the Genetics of Israeli Populations (Tel Aviv University, Israel) [19]. Lymphoblastoid cell lines were maintained in RPMI-1640 medium supplemented with 10% FBS, 2 mM glutamine, 100 units/mL penicillin and 100 μg/mL streptomycin. All reagents were purchased from Biological Industries, Kibbutz Beit Haemek, Israel. Cells were maintained in a humidified 5% CO_2_ atmosphere at 37 °C. Before hormonal treatments, USC cells were kept in serum-free (starvation) media for 24 h, after which they were treated with 50 ng/mL of IGF1 (PeproTech Ltd., Rocky Hill, NJ, USA) or insulin (Sigma-Aldrich, St. Louis, MO, USA) for 18 h. All experiments were carried out in triplicates.

### 2.2. Quantitative Real-Time Polymerase Chain Reaction (qPCR)

Total RNA was prepared from endometrial cancer cell lines using the Trizol reagent (ThermoFisher Scientific, Waltham, MA, USA). Two-hundred ng of total RNA was reverse transcribed using the Superscript First-Strand Synthesis System for cDNA synthesis by PCR (ThermoFisher Scientific). Quantitative real-time PCR was performed using Faststart Universal SYBR Green Mix (Sigma-Aldrich). An ABI Prism 7000 Sequence Detection System was employed for the qPCR. The number of PCR cycles to reach the fluorescence threshold is the cycle threshold (Ct). Each cDNA sample was tested in triplicate and the mean Ct values are reported. For each reaction, a “no template” sample was included as a negative control. Fold differences were calculated using the 2ΔΔCt method [38]. The primers used for OR5H2 mRNA detection were: sense, 5′-CACAGGACTTACATATCAGCCAG-3′; and antisense, 5′-AGACCAAGGTTCCACACAATAGT-3′. The size of the PCR band was 93 bp. For control purposes, the levels of GAPDH mRNA were measured.

### 2.3. Western Blot Analysis

Cells were treated with IGF1 or insulin for 24 h, after which they were washed with ice-cold phosphate-buffered saline (PBS) containing 5 mM EDTA and centrifuged at 1100 rpm. Lysis buffer was added and the cells were incubated on ice for 20 min. Cells were centrifuged at 13,000 rpm for 10 min and protein concentration was determined by the Bradford method. Samples were electrophoresed through 10% or 7.5% SDS-PAGE, followed by blotting of the proteins onto nitrocellulose membranes. After blocking with 5% skim milk, blots were incubated overnight with antibodies against OR5H2 (#102-12846, RayBiotech, Norcross, GA, USA), HSP70 (#7298, Santa Cruz Biotechnology, Santa Cruz, CA, USA) and total p53 (mixture of #126 and #98, Santa Cruz Biotechnology). In addition, membranes were incubated with antibodies against IGF1R (#3027), total-ERK (#9102), phospho-ERK (#9101S), total-AKT (#9272), phospho-AKT (#9271S) and phospho-p53 (#9284). Antibodies were obtained from Cell Signaling Technology (Danvers, MA, USA). Horseradish peroxidase (HRP)-conjugated secondary antibody, donkey anti-mouse and goat anti-rabbit were purchased from Jackson ImmunoResearch Laboratories (West Grove, PA, USA).

### 2.4. Small-Interfering RNA (siRNA) OR5H2 Knockdown

For OR5H2 knockdown, siRNA against human OR5H2 SMARTpool and non-targeting (NT) pool (Dharmacon Inc, Lafayette, CO, USA) were used. siRNAs were transfected using INTERFERin^TM^ (Polyplus Transfection, Illkirch, France). Briefly, the cells were seeded into 6 cm plates the day before transfection, and 7.5 nM (USPC1 cells) or 10 nM (USPC2 cells) of siRNA and 15 μL of INTERFERin^®^ was used for each transfection. OR5H2 knockdown was tested by immunoblot analysis.

### 2.5. Proliferation Assays

To assess the proliferation rate, cells were seeded onto 6-cm plates (1 × 10^4^ cells/well) and, after 24 h, were transfected with siRNA against OR5H2 or NT for 72 h (USPC1) or 48 h (USPC2) in triplicates. Cells were counted using a Cellometer Auto X4 Cell Counter (Nexcelom Bioscience, Lawrence, MA, USA) before and after transfection.

### 2.6. Cell Cycle Analysis

Twenty-four hours prior to siRNA addition, the cells were split into 6-cm plates (1 × 10^4^ cells/plate). After 24 h, the transfection with siRNA or NT was started. After an additional 72 h or 48 h (according to each cell line’s optimal siRNA concentration and time point) the cells were washed with PBS, trypsinized, centrifuged, re-suspended in 70% ethanol and stored at 4 °C. Prior to analysis, the cells were permeabilized and washed with PBS before addition of DAPI dye. The stained cells were analyzed using a CytoFLEX4 System (Beckman Coulter Life Sciences, Indianapolis, IN, USA).

### 2.7. Co-Immunoprecipitation (Co-IP) Assays

Total lysates (500 μg protein) were diluted in 200 μL IP buffer [1% Triton X-100, 150 mM NaCl, 20 mM Tris buffer (pH 7.5)] in the presence of proteases and phosphatases inhibitors and immunoprecipitated with anti-IGF1R β-subunit (C20, Santa Cruz Biotechnology) for 18 h at 4 °C. At the end of the incubation, the Protein A/G plus agarose beads were added for 2 h at 4 °C. The immunoprecipitates were electrophoresed and immunoblotted with antibodies against IGF1R β-subunit or OR5H2. Total lysates were loaded as input co-IP control for each sample.

### 2.8. Animal Studies

The generation of the GHRKO and bGH transgenic mouse models were previously described [1,2]. Mice were housed in a facility with 12-h light–dark cycles and free access to food and water. All animal procedures were approved by the Institutional Animal Care and Use Committee of the NYU School of Medicine (Assurance number A3435-01, USDA license No. 465), and conform to the Animal Research: Reporting of In Vivo Experiments (ARRIVE) guidelines (http://www.nc3rs.org.uk/arrive-guidelines, accessed on 4 March 2020). The approval date for the ethical authorization was 2 April 2020. Expiration date: 4 April 2023. RNA from the kidneys, uteri and ovarian tissues was extracted with an RNAeasy plus Mini Kit (Qiagen, Hilden, Germany). Thirty mg of tissue sample was homogenized with a Tissue lyser II at 30,000 rpm. The homogenized tissues were then applied to gDNA Eliminator Mini Spin Columns and washed extensively before eluting RNA in water. Finally, 30 μL of DEPC-dH_2_O was added to the eluted RNA. For cDNA synthesis, 1 μg of RNA was employed using the Superscript III First Strand kit (ThermoFisher Scientific).

### 2.9. Statistical Analysis

The statistical significance of the differences between groups was assessed by Student’s t-test (two samples, equal variance). Scanning densitometry analyses were evaluated using TINA imaging analysis software. The signal intensities of the proteins were normalized to the corresponding HSP70 signals. Data are presented as mean ± SEM of two or three independent experiments. *p* values < 0.05 were considered statistically significant.

## 3. Results

### 3.1. Identification of OR5H2 as a Target for IGF1 Action

**Figure 1:**
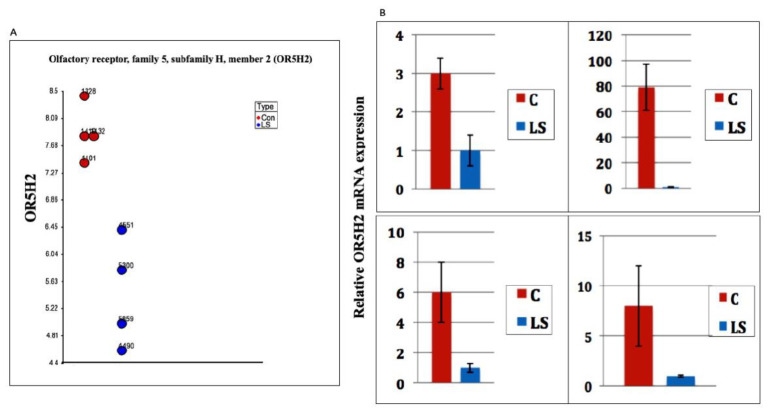
Expression of OR5H2 mRNA in the LS- and control-derived lymphoblastoid cell lines. (**A**) Dot-plot analysis of OR5H2 mRNA in lymphoblastoid cultures was conducted as described in Lapkina-Gendler et al. [19]. Dots denote individual LS patients (blue dots) and age-, gender- and ethnic-origin-matched controls (red dots). Numbers near each dot are code numbers. The y-axis denotes arbitrary gene expression values as measured in gene arrays. (**B**) Total RNA was obtained from four individual LS and four control lymphoblastoid cell lines and OR5H2 mRNA levels were measured by qPCR. The y-axis denotes the fold-change in gene expression between individual LS patients (blue bars) and matching controls (red bars). The bars represent the mean ± SEM of three independent cultures.

Genome-wide profiling of LS patients identified *OR5H2* as the top downregulated gene in this type of congenital IGF1 deficiency [19,20]. Specifically, OR5H2 mRNA levels were 5.8-fold lower in the LS- than in the control-derived lymphoblastoid cell lines (Figure 1A). To validate the differences in gene expression between LS patients and the healthy controls identified in genomic analyses, qPCR was performed. Levels of OR5H2 mRNA were markedly reduced in four pairs of the LS-derived lymphoblastoid cell lines compared to gender-, age- and ethnic-origin-matched controls (Figure 1B). These measurements confirm gene array data and suggest that reduced OR5H2 levels in LS might result from the relaxation of IGF1 stimulation.

### 3.2. Regulation of OR5H2 Gene Expression by IGF1 and Insulin in Endometrial Cancer Cells

**Figure 2:**
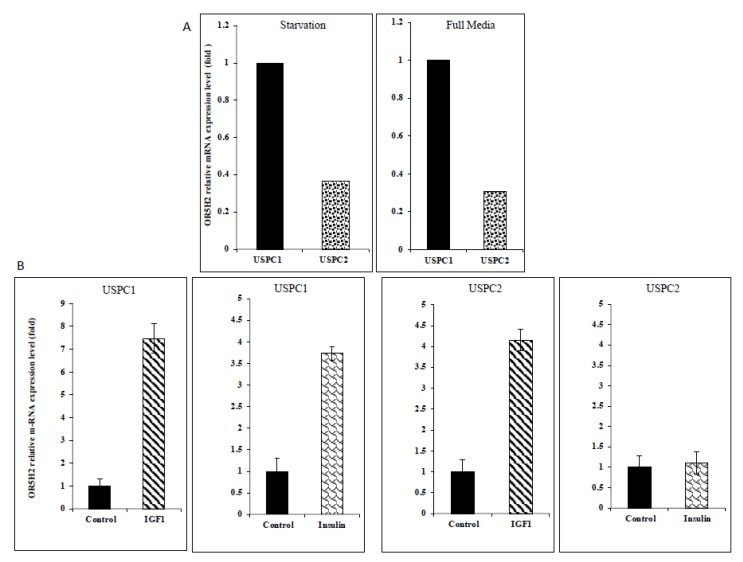
Effect of IGF1 and insulin on OR5H2 mRNA levels in USPC1 and USPC2 endometrial cancer cell lines. (**A**) USPC1 and USPC2 cells were kept in serum-free (starvation) or serum-containing (full) media for 24 h, after which total RNA was extracted and steady-state OR5H2 mRNA levels were measured by qPCR. (**B**) USPC1 and USPC2 cells were serum-starved for 24 h, after which they were treated with IGF1 or insulin (50 ng/mL) for 24 h. At the end of the incubation period, cells were harvested and the OR5H2 and GAPDH mRNA levels were quantitated by qPCR. A value of 1 was given to the normalized value in control cells. Bars are mean ± SEM of three independent experiments, each performed in triplicate.

To investigate the potential role of the *OR5H2* gene in endometrial cancer biology, we measured the basal OR5H2 mRNA levels in the USPC1 and USPC2 cell lines in the initial experiments. As described in *Materials and Methods*, cells were derived from USC patients who experienced rapid tumor growth after primary surgical debulking. The mutational analysis of the *p53* gene identified two polymorphisms in the USPC1 cell line (intron 3: c.96+41del16bp; exon 4: c.97-29C > A) and a homozygote C to T nucleotide exchange (exon 5: position c.493) in the USPC2 cell line that results in the formation of a stop codon at position p.165 [36]. As shown in Figure 2A, USPC1 cells express higher levels of OR5H2 mRNA than USPC2 cells under both serum-containing and serum-free growing conditions.

**Figure 3:**
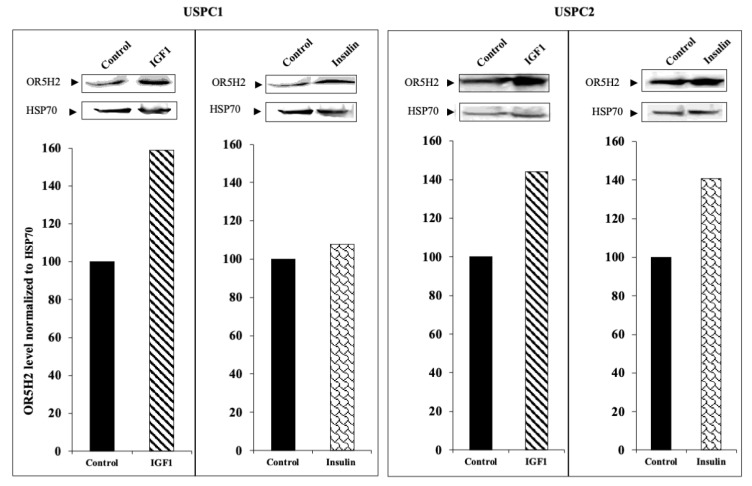
Effect of insulin and IGF1 on OR5H2 protein levels in USPC1 and USPC2 cell lines. USPC1 and USPC2 cell lines were starved overnight and then incubated with IGF1 or insulin (50 ng/mL) for an additional 24 h. Cells were lysed and levels of OR5H2 were measured by Western blots. Levels of HSP70 were measured as a loading control. Bars represent densitometric values of OR5H2 levels normalized to the corresponding HSP70 band. A value of 100% was given to the normalized value in untreated cells. Results of a representative experiment, repeated three times with similar results, are shown.

To assess the effect of IGF1 and insulin on *OR5H2* expression, the USPC1 and USPC2 cells were grown in a serum-free medium for 24 h and then treated with IGF1 or insulin (50 ng/mL) for an additional 24 h. The relative OR5H2 mRNA expression was evaluated by qPCR and normalized to the internal control GAPDH mRNA. IGF1 enhanced the OR5H2 mRNA levels in the USPC1 and USPC2 cells by 7.3- and 4.2-fold, respectively. Insulin stimulated expression only in the USPC1 cell line (3.7-fold increase) (Figure 2B). To establish whether the mRNA changes were associated with corresponding changes at the protein level, OR5H2 was measured by Western blots. Both hormones stimulated the OR5H2 protein levels in both cell lines, although the effect of insulin in the USPC1 cells was very small (Figure 3). Taken together, the effect of IGF1 on the OR5H2 mRNA and protein levels was stronger than that of insulin in both cell lines. Data confirm that the *OR5H2* gene is a downstream target for IGF1/insulin stimulatory activity.

### 3.3. Effect of OR5H2 Knockdown on the IGF1R Signaling Pathway

**Figure 4:**
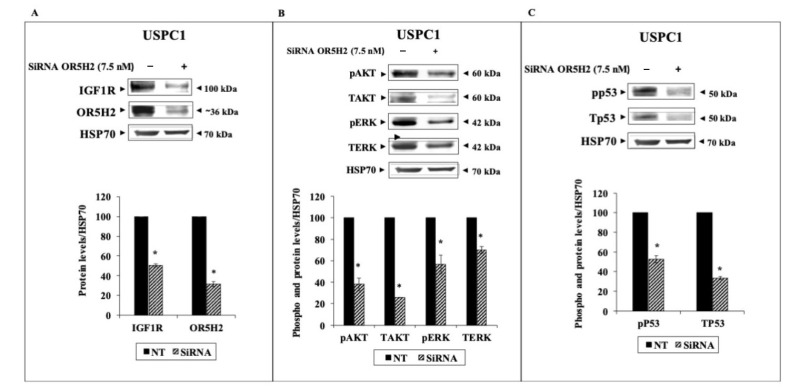
Effect of OR5H2 knockdown on downstream signaling proteins in USPC1 cells. USPC1 cells were transfected with 7.5 nM of OR5H2 siRNA (or non-targeting siRNA) for 72 h. At the end of the incubation period cells were harvested and lysates (130 mg) were analyzed by Western blots for IGF1R (**A**), total and phospho-AKT and ERK (**B**), and total and phospho-p53 (**C**) levels. Scanning densitometric analyses are presented for each protein. *, *p* < 0.01 versus respective control.

**Figure 5:**
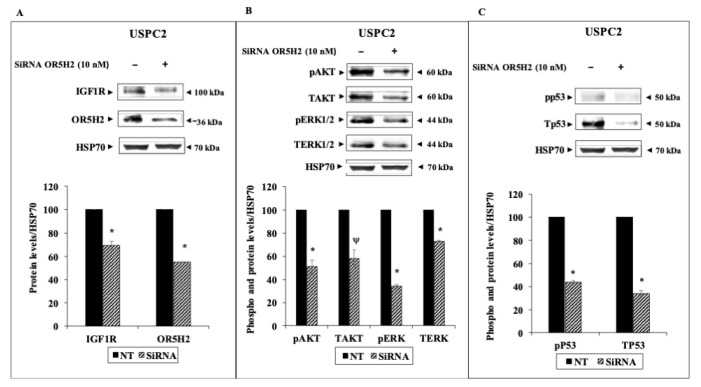
Effect of OR5H2 knockdown on downstream signaling proteins in USPC2 cells. USPC2 cells were transfected with 10 nM of OR5H2 siRNA (or non-targeting siRNA) for 48 h, after which cells were harvested and evaluated for IGF1R (**A**), phospho- and total-AKT and ERK (**B**), and total and phospho-p53 (**C**) levels. *, *p* < 0.01 *versus* respective control. Ψ *p* < 0.05 versus respective control.

To examine the effect of OR5H2 knockdown on key downstream mediators, USPC1 and USPC2 cells were transfected with a specific siRNA against OR5H2 (or non-targeting siRNA, for control purposes), as described in *Materials and Methods*. Calibration experiments were conducted to determine the optimal siRNA concentrations and transfection times for each cell line. Briefly, the calibration was done using two different doses (7.5 and 10 nM) and silencing was evaluated at 48 h (USPC2) or 72 h (USPC1) for each concentration (not shown). Western blots revealed a decrease in IGF1R levels (49.5% and 30.5% reductions in the USPC1 and USPC2 cells, respectively) upon *OR5H2* gene silencing (70% decrease in OR5H2 expression in USPC1 and 45% in USPC2) (Figure 4A,B and Figure 5A,B). In addition, marked decreases in the total and phosphorylated levels of AKT and ERK1/2 were noticed in both cell lines. Similarly, the total- and phospho-p53 were reduced upon OR5H2 knockdown in the USPC1 cells (Figure 4C). On the other hand, the total p53 was very low in the USPC2 cell line due to the mutation described above (Figure 5C). Of interest, Western blots using anti-phospho-p53 detected a phosphorylated p53 band whose intensity was diminished upon OR5H2 knockdown. Taken together, the data indicate that OR5H2 abrogation leads to significant the downregulation of IGF1R expression and activation, with ensuing reductions in the typical downstream targets.

### 3.4. Co-Immunoprecipitation of IGF1R and OR5H2

**Figure 6:**
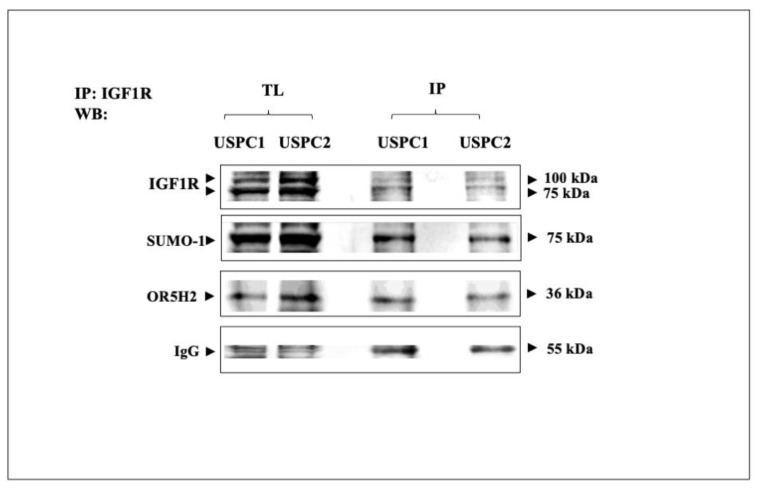
Analysis of physical interactions between IGF1R and OR5H2. USPC1 and USPC2 cells were lysed and immunoprecipitated with anti-IGF1R. Precipitates were electrophoresed through 10% SDS-PAGE, blotted onto nitrocellulose filters, and immunoblotted with anti-OR5H2, anti-IGF1R or anti-Sumo1. IP, immunoprecipitation; WB, Western blotting; TL, total lysate. Results of a typical experiment repeated twice with similar results are shown.

To investigate whether the functional interactions between IGF1R and OR5H2 were correlated with physical interactions between both proteins, co-IP experiments were performed. The USPC1 and USPC2 cells were lysed and immunoprecipitated with anti-IGF1R and the precipitates were incubated with protein A/G beads for 2 h. The precipitates were electrophoresed through 10% SDS-PAGE, transferred to nitrocellulose membranes and blotted with anti-IGF1R, anti-Sumo1 or anti-OR5H2. The results obtained showed that immunoblotting with anti-OR5H2 identified the 36-kDa protein in the anti-IGF1R immunoprecipitates (Figure 6). Likewise, anti-Sumo1 identified the 75-kDa band in the IGF1R immunoprecipitates. These results indicate that the functional interactions between IGF1R and OR5H2 are most probably correlated with a physical interaction between both proteins. Furthermore, our results indicate that IGF1R is sumoylated in both cell lines. However, it is still unknown whether IGF1R sumoylation is a pre-requisite for the interaction between IGF1R and OR5H2.

### 3.5. Effect of OR5H2 Knockdown on Endometrial Cell Proliferation

**Figure 7:**
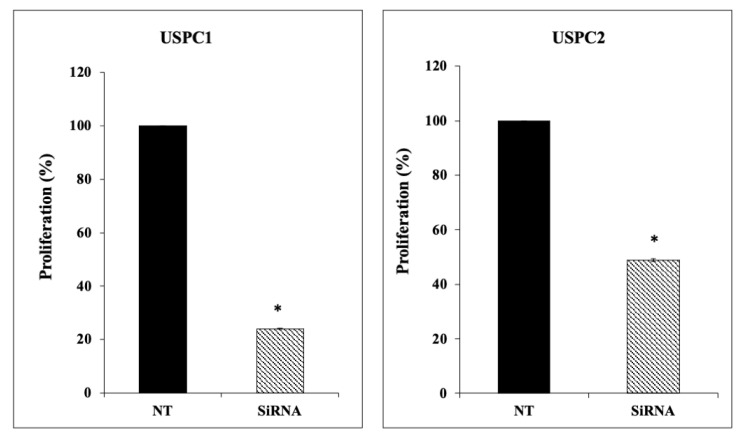
Effect of OR5H2 knockdown on cell proliferation. USPC1 and USPC2 cells (1 × 10^4^ cells/well) were plated on 6-cm plates in serum-containing media and, after 24 h, *OR5H2* gene expression was abolished as described in *Materials and Methods*. Cell proliferation was measured after 72 h (USPC1) or 48 h (USPC2) using a Cellometer cell counter. Results of a representative experiment repeated three times with similar results are shown. *, *p* < 0.01 versus respective control.

Next, we investigated the effect of OR5H2 expression on endometrial cancer cell proliferation. To this end, the *OR5H2* gene was knocked down as described above, followed by cell counting. The results obtained indicate that OR5H2 siRNA-transfected cells showed a significant reduction in cell proliferation compared to the controls in both endometrial cell lines (Figure 7). Thus, reductions of 76% and 51% were seen in the USPC1 and USPC2 cells, respectively. These results suggest that OR5H2 displays proliferative activities both in the presence of a wild-type (USPC1 cells) or a mutant (USPC2 cells) *p53* gene.

### 3.6. Effect of OR5H2 Knockdown on Cell Cycle Dynamics

**Figure 8:**
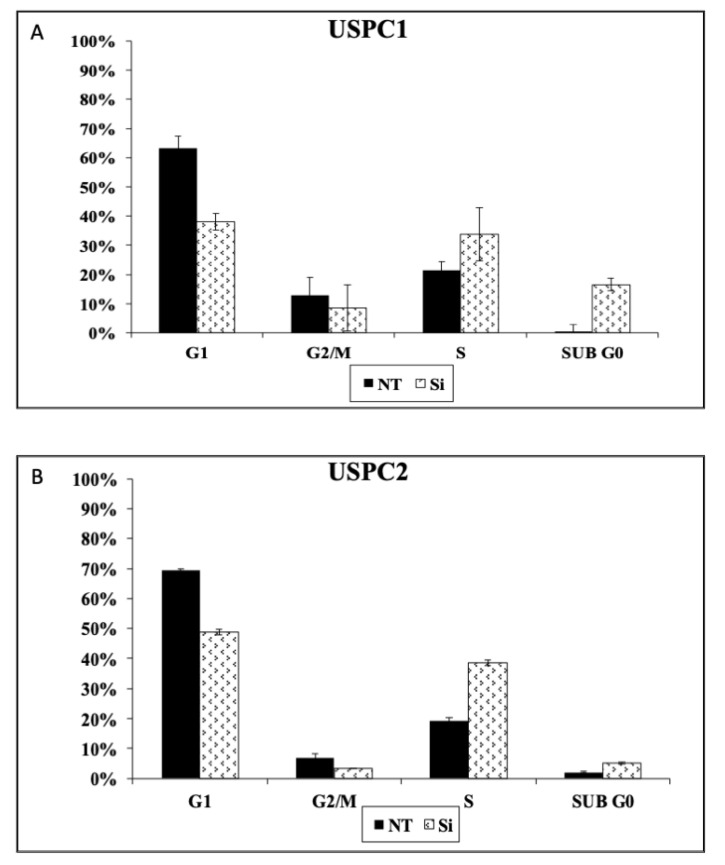
Effect of OR5H2 knockdown on cell cycle progression. USPC1 (**A**) and USPC2 (**B**) cells were seeded in quadruplicate dishes and transfected with OR5H2 siRNA or non-targeting siRNA for 72 h or 48 h, respectively. Cell cycle distribution was assessed by FACS analysis. The figure shows the results of a representative experiment repeated three times with similar results.

To evaluate the effect of OR5H2 on cell cycle progression, the gene was silenced as described in *Materials and Methods*, after which cells were tripsinized, centrifuged, resuspended in 70% ethanol and stored at 4 °C. Prior to analysis, cells were permeabilized before adding the DAPI dye. Flow cytometry analyses revealed a significant increase in the proportion of apoptotic (Sub G0) USPC1 cells following OR5H2 knockdown. In addition, silencing led to a reduction of approximately 10% in the portion of cells at the G2/M phase, a 40% reduction in cells at the G1 phase and an approximately 20% increase in cells at the S phase (Figure 8A). In the USPC2 cells, OR5H2 silencing led to a 5-fold increase in the proportion of apoptotic cells compared to the control. In addition, there were reductions of 20.7% and 3.3% in the G1 and G2/M phases, respectively, and a 19.2% increase in the proportion of cells in the S phase (Figure 8B). Taken together, the results show that the *OR5H2* gene affects cell cycle progression in a similar fashion in both wild-type and mutant-p53-expressing cells.

### 3.7. Animal Studies

**Figure 9:**
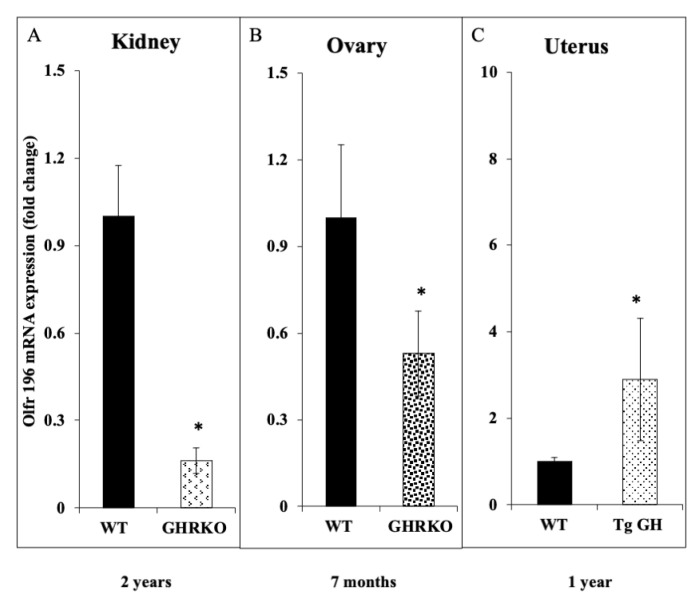
Expression of olfr196 mRNA in GHRKO and GH transgenic mice. Total RNA was prepared from the kidneys (**A**), ovaries (**B**) and uteri (**C**) of GHRKO (**A**,**B**) or GH transgenic (**C**) mice, or control littermates, and the olfr196 mRNA levels were measured by qPCR. Bars represent mean ± SD of 3–6 independent samples. *, *p* < 0.05 versus respective control.

To corroborate the dependence of *OR5H2* gene expression on the circulating IGF1 levels, the expression of olfr196 mRNA (the mouse orthologue of OR5H2) was measured in two different animal models: (1) the GHRKO (‘Laron’) mouse, an animal model of IGF1 deficiency, and (2) bGH transgenic mouse, overexpressing the *bGH* gene. The olfr196 mRNA levels were reduced by ~6.2-fold in the kidneys of 2-year-old GHRKO mice compared to the wild-type littermates (Figure 9A). In the ovaries, the olfr196 mRNA levels were reduced by 1.9-fold in the 7-month-old GHRKO mice compared to the controls (Figure 9B). Finally, the olfr196 mRNA levels were 3.3-fold higher in uteri of the bGH transgenic mice than in the controls (Figure 9C). Hence, the data support the notion that the *OR5H2/olfr196* gene is a physiological target for IGF1 action in a number of organs and at various developmental stages.

## 4. Discussion

The role of the GH-IGF1 signaling axis in the initiation and progression of cancer has been well established [39]. Consistent with its potent antiapoptotic and prosurvival actions, elevated systemic levels of IGF1 seem to confer an enhanced cancer risk [40,41,42]. This linkage is particularly compelling in a number of malignancies associated with endocrine or hormonal function, including breast and prostate tumors. In the case of endometrial cancer, several studies reported a correlation between components of the IGF system and uterine cancer risk [35,43]. In addition to the impact of circulating IGF1 on the hazard of developing a tumor, the expression and ligand-dependent activation (phosphorylation) of the IGF1R are regarded as key prerequisites for oncogenic transformation. Combined, the above findings provide a foundation to the postulate that the IGF1R represents a candidate therapeutic target in oncology [7]. The identification of biomarkers that may help in selecting patients and monitoring response to IGF1R-directed therapy is of cardinal importance in gynecologic oncology [44].

The epidemiological observation that patients with LS, the best characterized entity under the spectrum of the congenital IGF1 deficiencies, are protected from cancer development is of major clinical relevance [16,17]. The linkage between life-long diminished IGF1 dosages and cancer evasion emphasizes the central role of this growth factor in the etiology of cancer [18]. Genomic profilings conducted on LS-derived lymphoblastoid cells identified sets of genes that are either under- or over-represented in LS, and that might provide a mechanistic explanation for cancer protection in this condition [19].

The present study identified the *OR5H2* gene as a new downstream target for IGF1 action. OR5H2 emerged as the top downregulated gene in our genomic analyses, its mRNA levels being ~5.8-fold lower than in the age-, gender- and ethnic-origin-matched healthy controls. Based on these findings, we postulated that the *OR5H2* gene expression is under positive IGF1 regulation. This hypothesis was confirmed by in vitro experiments showing that IGF1 stimulated the OR5H2 mRNA levels by 4.2- to 7.3-fold in two endometrial cancer cell lines. The enhanced mRNA values were associated with corresponding changes in OR5H2 protein levels, although the extent of the increase in protein levels was lower than that seen at the mRNA level. Similarly, the insulin treatment stimulated OR5H2 mRNA levels in the USPC1, but not the USPC2, cells. Further corroboration of the role of *OR5H2* as a target for positive regulation by IGF1 was provided by animal studies showing that olfr196 mRNA levels were markedly reduced in the kidney and ovaries of GHRKO mice, a validated model of IGF1 deficiency. On the other hand, olfr196 mRNA levels were elevated in the uteri of GH transgenic mice.

While IGF1 stimulates OR5H2 mRNA and protein levels, it is not yet clear whether this effect is achieved at the level of *OR5H2* gene transcription or, alternatively, via the enhanced stability of the transcript. However, inspection of the *OR5H2* promoter region demonstrated the presence of a number of canonical binding sites for transcription factors Sp1, C/EBP-α, AP-1, c-jun, c-fos, etc. (https://www.genecards.org/cgi-bin/carddisp.pl?gene=OR5H2 accessded on 4 March 2021). Future molecular and bioinformatic analyses are expected to unravel the role of these *cis*-elements as putative IGF1-responsive elements.

Of interest, OR5H2 knockdown in both endometrial cancer cell lines led to marked reductions in IGF1R protein levels and in the total and phosphorylated levels of the AKT and ERK1/2 cytoplasmic mediators. Data is consistent with a feedback regulatory loop model in which, on one hand, IGF1/insulin govern the *OR5H2* gene expression and, on the other hand, OR5H2 regulates the *IGF1R* gene expression and action. Despite the fact that basal levels of OR5H2 were higher in the USPC1 (expressing a wild-type p53) than in USPC2 (expressing a mutated p53) cells, the OR5H2 knockdown led to similar effects in terms of proliferation and cell cycle dynamics in both cell lines. Hence, the proliferative properties of OR5H2 seem to be independent of the p53 mutational status.

To the best of our knowledge, the role of ORs in general, and OR5H2 in particular, in endometrial cancer has not yet been investigated. Other ORs have been identified as tumor promoters in a number of malignancies. For example, OR7C1 was reported to have essential roles in the maintenance of colon cancer initiating cells [26]. Flow cytometry indicated that OR7C1-positive cells showed higher tumorigenicity than OR7C1-negative cells, suggesting that OR7C1 is a novel functional marker for colon cancer initiation. Furthermore, a high expression of OR7C1 correlated with poor prognosis in colorectal cancer patients. Likewise, the high expression of OR51E1 in somatostatin receptor-negative lung carcinoids and in small intestine neuroendocrine carcinomas makes OR51E1 a potential novel diagnostic biomarker in these types of tumor [27,28]. Of translational relevance, recent studies have shown that galllein, a Gbg subunit signaling inhibitor, was capable of inhibiting the metastatic spread of prostate cancer cells expressing OR51E2 and, exposed to b-ionone, its odorant ligand [45].

The complexity of the olfactory receptor superfamily, however, makes analyses of the role of specific components very difficult [21,22]. While our data and that of others suggest a proliferative, anti-apoptotic role for ORs, other studies are consistent with a tumor suppressing role for members of this family. Thus, ectopically expressed OR51E1 and OR51E2 suppressed prostate cancer cells growth in association with the upregulation of cytostatic and cell death markers, including p27, p21 and p53, and enhanced annexin V staining [46]. Taken together, ORs display organ-specific, concentration-dependent bioactivities. Generalizations on their mechanisms of action are discouraged.

As alluded to above, the identification of biomarkers that can contribute to the successful delivery of IGF1R-directed therapies is of major importance in clinical oncology [47,48,49]. The identification of OR5H2 as an IGF1-dependent, mitogenic factor in endometrial cancer might help in these efforts [44]. The fact that OR5H2 is under-represented in LS, a genetic condition associated with cancer protection, provides further support to the concept that OR5H2 has an important role in the biology of cancer. Future studies will address the question whether co-targeting of IGF1R along with OR5H2 might lead to better clinical outcomes [36,50].

In summary, our data identified a previously unrecognized connection between the IGF1 axis and a family of olfactory receptors. OR5H2 emerged as a new target for positive regulation by IGF1, with potential relevance in endometrial cancer biology. The clinical significance of our findings, including the analysis of OR5H2 expression in tumors and its correlation with clinical parameters, should be investigated in the clinical setting. Finally, the mechanistic aspects of the connection between the IGF1 and ORs signaling pathways must be further dissected.

## Data Availability

Not applicable.
